# Selenoprotein S: a therapeutic target for diabetes and macroangiopathy?

**DOI:** 10.1186/s12933-017-0585-8

**Published:** 2017-08-10

**Authors:** Shan-shan Yu, Jian-ling Du

**Affiliations:** Department of Endocrinology, The First Affiliated Hospital of Dalian Medical University, Dalian, 116011 Liaoning China

**Keywords:** Selenoprotein S, Diabetes mellitus, Atherosclerosis, Cardiovascular diseases, Single nucleotide polymorphism

## Abstract

Inflammatory response, oxidative stress, and endoplasmic reticulum (ER) stress are important pathophysiological bases of the occurrence and development of diabetes mellitus (DM) and macroangiopathy complications. Selenoprotein S (SELENOS) is involved in the regulation of these mechanisms; therefore, its association with DM and macroangiopathy has gradually received attention from scholars worldwide. SELENOS has different biological functions in different tissues and organs: it exerts antioxidant protection and has anti-ER stress effects in the pancreas and blood vessels, while it promotes the occurrence and development of insulin resistance in the liver, adipose tissue, and skeletal muscle. In addition, studies have confirmed that some SELENOS gene polymorphisms can influence the inflammatory response and are closely associated with the risk for developing DM and macroangiopathy. Therefore, comprehensive understanding of the association between SELENOS and inflammation, oxidative stress, and ER stress may better elucidate and supplement the pathogenic mechanisms of DM and macroangiopathy complications. Furthermore, in-depth investigation of the association of SELENOS function in different tissues and organs with DM and macroangiopathy may facilitate the development of new strategies for the prevention and treatment of DM and macrovascular complications. Here, we summarize the consensus and controversy regarding functions of SELENOS on currently available evidence.

## Background

In 2016, the World Health Organization (WHO) declared that diabetes mellitus (DM) had become the eighth most prevalent cause of disease-related mortality. Its onset is no longer mainly observed in economically developed areas; in recent decades, the number of patients has increased significantly in middle- and low-income countries. Therefore, DM has been confirmed to be a “global” public health issue [1]. The prevalence of adult DM patients globally increased from 4.7% in 1980 to 8.5% in 2014 [1]. In addition, 84% of DM patients die of complicated cardiovascular disease (CVD) and stroke [2]. Therefore, investigation of key factors affecting the occurrence and development of DM and its macroangiopathy complications and the search for effective prevention and treatment strategies have become an important research mission for scholars.

Selenium (Se), a trace element, is a key component of selenoproteins involved in a wide range of functions, including redox homeostasis, inflammatory regulation, thyroid hormone metabolism, immunity, myocardial and tumoral diseases, and reproduction [3–6]. Moreover, it is well documented that Se is associated with glucose metabolism, and both over supplementation and deficiency of Se can be associated with type 2 DM (T2DM) risk, following a U-shaped curve [3, 7, 8]. Selenoproteins are important metabolites of dietary Se, fulfilling the catalytic effects of Se, and are characterized by a selenocysteine (Sec), a selenium-containing amino acid encoded from the stop codon UGA in the molecular structure [9–12]. To date, a total of 25 selenoproteins have been discovered in humans, among which glutathione peroxidase 1 (GPX1), iodothyronine deiodinase 3 (DIO3), selenoprotein P (SELENOP), selenoprotein S (SELENOS), and selenoprotein T (SELENOT) have been documented to correlate with glucose homeostasis [8, 12, 13].

Selenoprotein S (SELENOS), as a member of the selenoprotein family, was first discovered in the liver of *Psammomys obesus* (*P. obesus*) by Walder et al. [14] and was initially named Tanis. Subsequent studies showed that Tanis, AD-015, SelS, SELENOS, VIMP, and SEPS1 were the same protein [9, 12, 14–16]. SELENOS is involved in the regulation of inflammation, oxidative stress, and endoplasmic reticulum (ER) stress [14, 17–21]. The above pathophysiological reactions usually have reciprocal causations, mutually promote one another, and participate together in the occurrence and development of DM and macrovascular complications [22–25]. Therefore, the functions of SELENOS in DM and macroangiopathy have gradually received attention from scholars in China and other countries. This article aimed to elaborate the association between SELENOS and inflammation, oxidative stress, and ER stress and the function of SELENOS in the occurrence and development of DM and macroangiopathy to explore new strategies for the prevention and treatment of DM and its macrovascular complications.

## Expression pattern, structure, and distribution of SELENOS

**Fig. 1:**
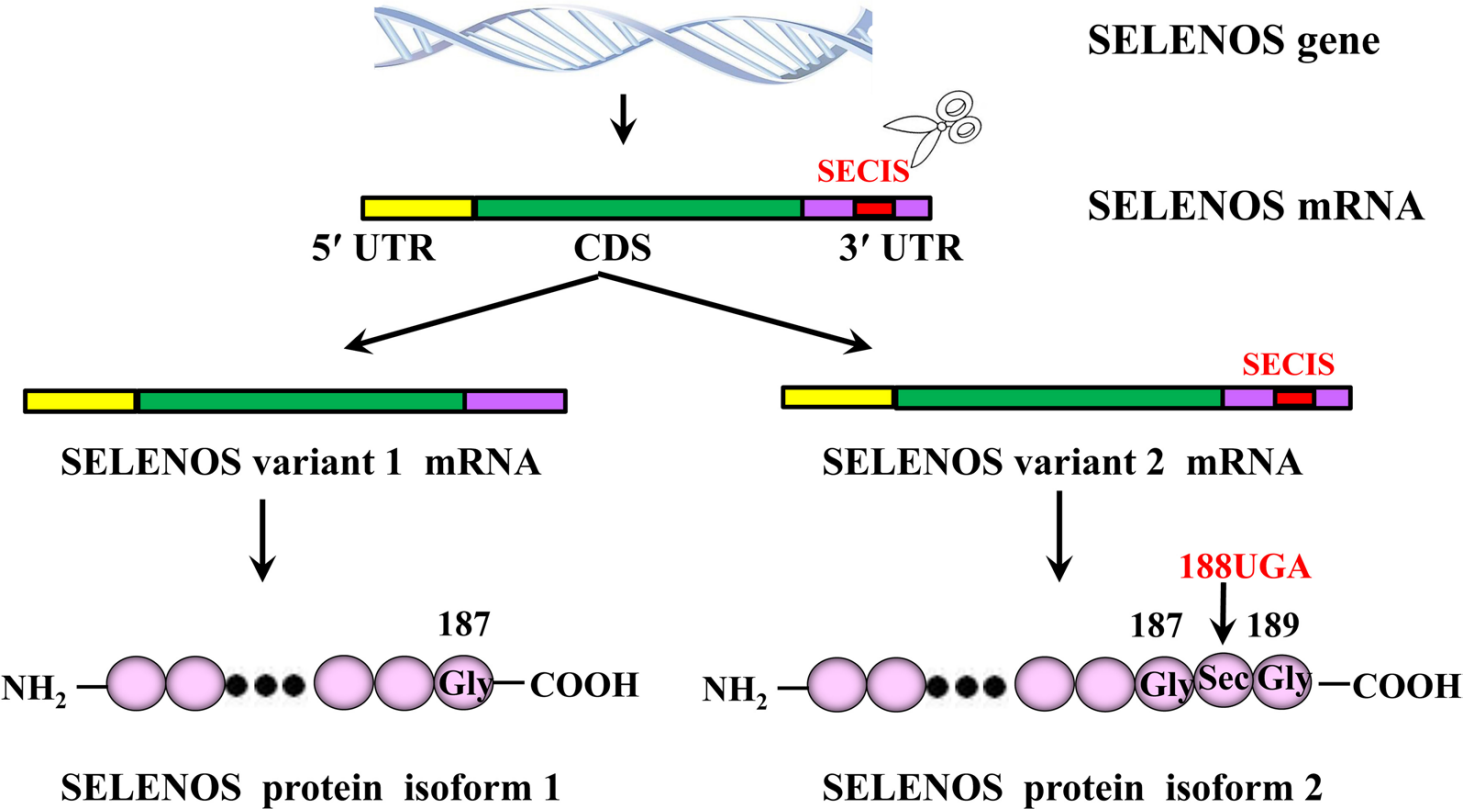
Schematic of the encoding of SELENOS mRNA variants and protein isoforms. The human SELENOS gene encodes two transcripts: variant 1 mRNA and variant 2 mRNA. They differ in a splicing event that removes a portion of the 3′UTR sequence containing a SECIS element from variant 1. The SECIS element is necessary to recode the ^188^UGA stop codon in the CDS of the SELENOS mRNA sequence to a selenocysteine. As a result, two SELENOS protein isoforms are expressed: the 187-aa isoform 1 from variant 1 and the 189-aa isoform 2 from variant 2. *UTR* untranslated region, *CDS* coding domain sequence, *SECIS* selenocysteine insertion sequence, *Gly* glycine, *Sec* selenocysteine

The human SELENOS gene (GenBank: NG_013322.1) is located on 15q26.3. Its primary transcript produces two types of transcription variants after selective splicing: SELENOS variant 1 (GenBank: NM_203472.2) and SELENOS variant 2 (GenBank: NM_018445.5). The main difference between these two transcripts is that the selenocysteine insertion sequence (SECIS) in the 3′untranslated region (3′UTR) of SELENOS variant 1 is spliced [26]. It is known that the SECIS plays an important role in selenoprotein biosynthesis. As a result of interactions of the SECIS with selenocysteine-transfer RNA, Sec-specific elongation factor, and SECIS-binding protein 2, the stop codon UGA in the coding domain sequence (CDS) of selenoprotein mRNA will not be recognized as the translation termination signal and will be encoded as Sec [27–30]. Therefore, the ^188^UGA codon in SELENOS variant 2 CDS can be normally encoded as Sec to produce the protein SELENOS isoform 2, with 189 amino acid residues. Because the SECIS in SELENOS variant 1 is spliced, the ^188^UGA in the mRNA CDS cannot be encoded as Sec and is recognized as the translation termination signal. Therefore, a short protein, SELENOS isoform 1, with 187 amino acid residues, is produced [26]. Recent studies have indicated that the short selenoprotein, SELENOS isoform 1, that is produced when the UGA cannot be encoded as Sec, is degraded through CRL2 ubiquitin ligase [31], suggesting that SELENOS exerts biological functions in the body in the form of SELENOS isoform 2 (Fig. 1).

SELENOS is a single-pass transmembrane protein with an ER region (1st–25th amino acid residues), a transmembrane region (26th–51st amino acid residues), and a cytoplasmic region (52nd–189th amino acid residues). The cytoplasmic region contains the valosin-containing protein (VCP)-interacting motif (VCP-interacting motif, VIM) composed of the 78th–88th amino acid residues, and there is a selenosulfide bond between ^174^Cys and ^188^Sec that has reductase activity [18].

In addition to localizing in the ER membrane [15, 18], SELENOS was confirmed to localize in the plasma membrane by Kryukov et al. [9]. Moreover, in 2013, Bubenik et al. [26] discovered a new subcellular localization of SELENOS in the perinuclear region of HepG2 liver cancer cells and confirmed that some of the SELENOS enriched in the perinuclear regions was located in Golgi bodies. In addition to intracellular localization, SELENOS has also been confirmed to be present in HepG2 cell culture supernatant [32, 33]. SELENOS is expressed in liver, skeletal muscle, lipid, pancreatic islet, kidney, central nervous system (hypothalamus, cerebellum, etc.), testis, and colon [14, 19, 32, 34–38]. Studies in recent years have also shown new histological distributions of SELENOS in spleen, blood vessel, and serum [33, 39–42].

## General bioactivity

### SELENOS and inflammation

When Walder et al. [14] first discovered SELENOS, they confirmed that it was a receptor for the acute inflammatory response protein, serum amyloid A (SAA); in addition, inhibition of SELENOS expression could up-regulate the expression of SAA in lipopolysaccharide (LPS)-induced HepG2 human liver cancer cells [43]. These results suggested a potential relationship between SELENOS and inflammatory reactions. Subsequently, Fradejas et al. [17] showed that SELENOS expression increased after induction of inflammatory injury in brain tissues of C57BL/6 mice, indicating that SELENOS was associated with inflammation. Next, in vitro induction of inflammatory injury in human and mouse astrocytes using LPS and interleukin-1β (IL-1β) also showed the up-regulation of SELENOS expression in corresponding cells. Furthermore, induction of SELENOS overexpression showed that it could reduce the expression of the inflammatory factors IL-1β and interleukin-6 (IL-6) in astrocytes that was stimulated by LPS. In contrast, inhibition of SELENOS expression further increased the expression of IL-1β and IL-6 stimulated by LPS. These results partially explained the molecular mechanism of the anti-inflammatory function of SELENOS. Liu et al. [44] established a cerebral ischemia/reperfusion injury model in SD rats to induce inflammation, and they found that neuronal cells in ischemic penumbra swelled, the cell density decreased, and SELENOS expression increased, which also confirmed the association between SELENOS and inflammation. In addition to inhibition of the expression of the inflammatory factors IL-1β and IL-6 by SELENOS, supplementation of selenium could also reduce the levels of tumor necrosis factor α (TNFα) and monocyte chemoattractant protein 1 (MCP 1) and reduce the secretion of interleukin-2 (IL-2) in primary porcine splenocytes [39, 45]. Selenium is the raw material for selenoprotein synthesis. Proper supplementation of selenium could increase SELENOS biosynthesis [45, 46], while inhibition of SELENOS expression could weaken the function of selenium supplementation on the reduction of IL-2 secretion in primary porcine splenocytes [39]. These results suggested that supplementation of selenium reduced the levels of TNFα, MCP-1, and IL-2 by increasing SELENOS biosynthesis, which further corroborated the molecular mechanism of the anti-inflammatory function of SELENOS.

Zhang et al. [35] found that the SELENOS gene in pigs had high homology with the human SELENOS gene (88%) and that the gene structures were very similar. In addition, the −601 to 398 region of the SELENOS gene promoter had an NF-κB binding site, a positive regulatory element for regulation of SELENOS gene expression. This binding site could increase SELENOS gene expression, and its sequence was confirmed to be the same as that in humans. In addition, a negative element that was suspected to regulate SELENOS gene expression in the −161 to 601 region of the SELENOS gene promoter was discovered. The above conclusion of Zhang et al. may be able to explain the study results of Su et al. [47], who injected LPS into the abdominal cavities of BALB/c mice to establish a septic mouse model. Their results showed that SELENOS expression in the livers of the mice increased, reached a peak value after 24 h of injury, gradually decreased afterward, and was reduced to normal levels after 72 h. Therefore, it was speculated that the increase of SELENOS expression at the early stage of injury may be associated with an up-regulation induced by NF-κB to protect tissues and cells from inflammatory injury. For the subsequent down-regulation of SELENOS expression, it was speculated that during the inflammatory injury process induced by LPS, some factors that interacted with the SELENOS negative regulatory region were produced to inhibit SELENOS expression, attenuate the protective function of SELENOS, and finally cause tissue and cell damage. Whether these regulatory transcription factors are specifically present in inflammation still awaits further in-depth studies.

Furthermore, studies in recent years on the association between SELENOS gene polymorphisms and inflammation have also become a hot research topic. Previously, Curran et al. [48] showed that the −105G→A single nucleotide variation (rs28665122) in the SELENOS gene promoter and the +3705G→A variation in the SELENOS gene were associated with serum TNFα, IL-6, and IL-1β and that the +5227C→T variation (rs4965373) in the SELENOS gene was associated with serum IL-1β, of which the association of the −105G→A variation was the most evident. Further studies showed that the −105G→A variation decreased the activity of the SELENOS promoter and reduced SELENOS expression in HepG2 liver cancer cells under the stimulation of ER stress, whereas inhibition of SELENOS expression increased TNFα and IL-6 production in RAW264.7 macrophages under ER stress. It is known that the −105G→A variation is located in the ER stress-response element (ERSE) of the SELENOS gene; therefore, it was speculated that this variation changed the ERSE sequence to affect SELENOS expression under the stimulation of ER stress and further influenced the levels of inflammatory factors [48]. However, the subsequent studies of Seiderer et al. [49] and Bos et al. [50] confirmed that the above three SELENOS single nucleotide polymorphisms (SNPs) were not associated with inflammatory factors. This controversial phenomenon may be explained by the study of Stoedter et al. [45], which confirmed that the selenium level in the body and gender could affect SELENOS expression and further affect cytokine levels. Therefore, these two confounding factors should be controlled in a study of SELENOS SNPs, and a stratified analysis should be performed based on the selenium levels in the body and gender. Bos et al. [50] also performed haplotype association analysis on three SNPs: SELENOS SNP rs28665122, rs4965814, and +6218 A→G variation (rs9874). The haplotype GAG was associated with serum IL-2, IL-6, interleukin 10 (IL-10), and granulocyte colony-stimulating factor (G-CSF). The above studies suggested that SELENOS SNPs play important roles in mediating inflammatory reactions. However, except for the study by Curran et al. [48], there is no report on the mechanism underlying the regulation of inflammatory reactions by SELENOS gene variations.

### SELENOS and oxidative stress

Christensen et al. [18] showed that SELENOS also had reductase activity. This reduction function was exerted through ^188^Sec and was maintained through the restoration of the selenosulfide bond between ^174^Cys and ^188^Sec. In other words, in the reduction process, ^188^Sec of SELENOS and substrates formed a mixed selenosulfide bond to cause substrate reduction. Next, through the restoration of the selenosulfide bond between ^174^Cys and ^188^Sec, SELENOS broke the mixed selenosulfide bond between SELENOS and substrates to maintain the activity of the SELENOS reductase. Liu et al. [51] showed that the reduction of the oxidized SELENOS to restore the selenosulfide bond between ^174^Cys and ^188^Sec depended on the function of thioredoxin (Trx), indicating that SELENOS was a Trx-dependent reductase. In addition, they also showed that SELENOS had peroxidase activity and could break down its substrate, hydrogen peroxide (H_2_O_2_), into H_2_O [51]. The above studies indicated that SELENOS was closely associated with oxidative stress. This viewpoint was confirmed in the studies of Zahia et al. [52] and Zeng et al. [43]. They found that H_2_O_2_ could up-regulate SELENOS expression in HEK293 human embryonic kidney cells, while inhibition of SELENOS expression could further aggravate the LPS-induced increase of reactive oxygen species levels in HepG2 human liver cancer cells, down-regulation of GPx expression, and inhibition of cell viability. These results suggested that SELENOS may have the function of protecting tissues and cells from oxidative stress-induced damage. Previously, Gao et al. [19] showed that in vitro induction of SELENOS overexpression in Min6 islet β cells could increase its resistance to H_2_O_2_ damage and enhance cell viability. Our study group induced SELENOS overexpression in human umbilical vein endothelial cells (HUVECs) and showed that SELENOS overexpression could significantly increase HUVEC viability after H_2_O_2_ stimulation, enhance the activity of superoxide dismutase (SOD), and reduce the production of methane dicarboxylic aldehyde (MDA) [41]. Gan et al. [53] showed that SELENOS-overexpressing porcine kidney PK15 cells could exert an antioxidant protection function through the reduction of reactive oxygen species (ROS) and the increase of glutathione (GSH) expression. Next, the study by Ye et al. [42] indicated that inhibition of SELENOS expression in vascular smooth muscle cells (VSMCs) further aggravated the reduction of the cell viability induced by H_2_O_2_, which was accompanied by the further increase of ROS and MDA levels and further reduction of the GPx activity. They also confirmed that the antioxidant protection function of SELENOS was associated with mitogen-activated protein kinase (MAPK) and c-JUN N-terminal kinase (JNK).

### SELENOS and ER stress

As an ER membrane protein, SELENOS plays an important role in the maintenance of the morphology and distribution of ER in cells [54]. In addition, it forms a complex with degradation in endoplasmic reticulum protein 1 (Derlin1)-ubiquitin ligase E3-p97ATPase to degrade unfolded or misfolded proteins in the ER, a process known as ER-associated protein degradation (ERAD) [20, 55–58]. Most recent studies have shown that selenoprotein K also participated in the composition of this complex [59]. Studies showed that the ER stress inducers tunicamycin (TM), thapsigargin (TG), and β-mercaptoethanol could up-regulate SELENOS expression in RAW264.7 macrophages, HepG2 liver cancer cells, HEK293T human embryonic kidney cells, and mouse astrocytes [21, 60–62], suggesting that SELENOS is involved in the regulation of ER stress. Overexpression of SELENOS attenuated the reduction in RAW264.7 macrophage viability and the increase in cell apoptosis induced by TG and TC [21], inhibition of SELENOS expression in mouse astrocytes further aggravated the reduction in cell viability induced by TG and TC [60], and inhibition of SELENOS expression in HepG2 liver cancer cells further aggravated cell apoptosis and further reduced the cell viability induced by β-mercaptoethanol [61]. These results indicated that SELENOS could protect cells from damage induced by ER stress. The above conclusion was explained in the studies of Kelly et al. [63] and Kim et al. [64]. They found that SELENOS overexpression could reduce the activity of the glucose-regulated protein 78 (GRP78) promoter, an ER stress marker protein, in HepG2 liver cancer cells and decrease the GRP78 protein expression induced by TC in HEK293T human embryonic kidney cells. Therefore, it was speculated that the up-regulation of SELENOS expression in the process of ER stress was to defend the further increase of GRP78 expression to exert its protective function in ER stress.

In addition to investigating the relationship between SELENOS and inflammation, Fradejas et al. [17] also analyzed the relationship between SELENOS and ER stress. They found that after human and mouse astrocytes were treated with the ER stress inducers TM and TG, SELENOS expression levels in these two cells were up-regulated and were significantly higher than that in the group with inflammatory injury induced by LPS and IL-1β. These results indicated that SELENOS was associated with ER stress and that the function of ER stress in the up-regulation of SELENOS expression was stronger than that of inflammatory stimulation. Fradejas et al. [17] further induced high SELENOS expression in astrocytes and showed that SELENOS could reduce the expression of the ER stress markers X-box binding protein 1 (XBP-1) and CCAAT/enhancer-binding protein homologous protein (CHOP), stimulated by TM and TG, which further corroborated the molecular mechanism underlying the protection of cells from ER stress by SELENOS. This conclusion was contradictory to the study of Speckmann et al. [38]. They inhibited SELENOS expression in LS174T colon cancer cells and showed that in the presence of the ER stress inducer TM, the expression of GRP78, XBP-1, and CHOP between the low SELENOS expression group and the normal control group were not different and the expression levels of the above ER stress markers in the low SELENOS expression group did not further increase. Subsequently, they repeated the above experimental processes in another two colon cancer cell lines, HT29 cells and Caco-2 cells, and also obtained the same results [38]. The difference in the types of studied cells may be the major reason for the inconsistent conclusions between these two studies. It was speculated that the participation of SELENOS in the regulation of ER stress was cell-type dependent; however, further studies are required for confirmation and explanation.

## SELENOS and glucose metabolism

### Evidence from human genome-wide association studies

Martínez et al. [65] compared the genotype frequencies of six types of SELENOS SNPs between type 1 DM (T1DM) patients (n = 310) and non-T1DM healthy controls (n = 550) in a Spanish population. The genotype frequencies of SELENOS SNPs rs11327127, rs28665122, rs4965814, rs12917258, rs4965373, and rs2101171 between these two groups were not different; the comparison after sex-stratification still did not show a difference. These results indicated that the above six types of SELENOS SNPs were not risk factors of T1DM. Olsson et al. [66] randomly selected 1236 subjects from the INTERGENE program, which analyzed the interplay between genetic susceptibility and environmental factors for the risk of chronic diseases in a Swedish population to analyze the relationship between three types of SELENOS SNPs and metabolic risk factors. The serum insulin levels and homeostasis model assessment of insulin resistance (HOMA-IR) between the group that carried SELENOS SNP rs4965373 and the group that did not carry this SNP were significantly different, while the above indicators between the groups that carried or did not carry rs28665122 and groups that carried or did not carry rs4965814 were not significantly different. The multiple linear regression analysis results after age, sex, and DM status were adjusted showed that SELENOS SNP rs4965373 correlated with the serum insulin level, SELENOS SNP rs4965814 correlated with the blood glucose level and HOMA-IR, and SELENOS SNP rs28665122 did not correlate with the above metabolic risk factors. Currently, there is still no study focusing on the association between SELENOS SNPs and DM. Case–controlled studies that involve more SELENOS SNPs, different races, and larger sample sizes still need to be performed (Table 1).

**Table 1:** Association between SELENOS SNPs and the risk of related diseases

No.	Rs number^a^	Location	Polymorphism [1/2]	Risk of related diseases	References
1	rs28665122	5′ near gene	G/A	Subclinical CVD in T2DM	[75]
				Intestinal type gastric cancer	[80]
				Non-small cell lung cancer	[84]
				Rheumatoid arthritis	[65]
				Hashimoto’s thyroiditis	[86, 87]
				Preeclampsia	[88]
				Spontaneous preterm birth	[89]
2	rs8025174	5′ near gene	C/A	Coronary heart disease	[72]
3	rs34713741	5′ near gene	C/T	Gastric cancer	[81]
				Colorectal cancer	[82, 83]
4	rs4965814	Intron	T/C	Diabetes	[66]
				Ischemic stroke	[73, 74]
				Subclinical CVD in T2DM	[75]
				CVD in T2DM	[75]
5	rs12917258	Intron	G/C	Subclinical CVD in T2DM	[75]
6	rs2009895	Intron	C/T	Hashimoto’s thyroiditis	[87]
7	rs4965373	3′UTR	G/A	Diabetes	[66]
8	rs9874	3′UTR	T/C	Ischemic stroke	[74]
9	rs28628459	3′UTR	T/C	Subclinical CVD in T2DM	[75]
				CVD in T2DM	[75]
10	rs7178239	3′ near gene	C/G	Ischemic stroke	[72]
				Subclinical CVD in T2DM	[75]
11	rs9806366	3′ near gene	C/T	CVD in T2DM	[75]

*CVD* cardiovascular disease, *T2DM* type 2 diabetes mellitus, *UTR* untranslated region

^a^Rs numbers are from the PubMed SNP database. 1 = major allele, 2 = minor allele

### Evidence from in vivo and in vitro studies

#### Liver

Walder et al. [14] showed that SELENOS expression in liver negatively correlated with blood glucose and serum insulin levels in type 2 DM (T2DM) and metabolic syndrome animal models in *P. obesus*. In addition, the inhibition of SELENOS expression by glucose in a concentration-dependent manner was also confirmed in in vitro cultured HepG2 liver cancer cells. Moreover, Gao et al. [19] confirmed that glucose at concentrations lower than 5 nM could increase SELENOS expression in HepG2 cells. These results showed that liver SELENOS was associated with glucose metabolism in the body. To further investigate the function of liver SELENOS in glucose metabolism, Gao et al. [34] induced high SELENOS expression in vitro in H4IIE liver cancer cells that had similar biological characteristics to hepatocytes in the body. The glycogenesis, glycogen content, and glucose uptake in H4IIE cells that expressed high SELENOS all decreased compared to those in the non-transfection and empty vector transfection groups, suggesting that liver SELENOS could decrease hepatic glycose utilization. In addition, they also found that high SELENOS expression attenuated the inhibitory function of insulin on phosphoenolpyruvate carboxykinase (PEPCK, a rate-limiting enzyme in gluconeogenesis) in H4IIE cells to increase gluconeogenesis, indicating that liver SELENOS could increase hepatic glucose output.

#### Adipose tissue

The expression of SELENOS in 3T3-L1 adipocytes was inhibited by glucose or insulin in a concentration-dependent manner [14]. Karlsson et al. [67] compared SELENOS mRNA expression in subcutaneous adipose tissues between T2DM patients (n = 10) and age- and body weight-matched healthy individuals (n = 11) and showed that there was no difference between these two groups. Patients in these two groups were subjected to a euglycemic-hyperinsulinemic clamp, and the expression of SELENOS mRNA in subcutaneous adipose tissues increased in patients of the T2DM group (1.67-fold), with no significant change in the healthy control group, suggesting that high insulin could increase SELENOS expression in subcutaneous adipose tissues in T2DM patients. The Pearson correlation analysis results showed that SELENOS expression in subcutaneous adipose tissues positively correlated with serum SAA. Next, our study group analyzed the expression of SELENOS mRNA in omental adipose tissues between T2DM patients (n = 10) and non-DM individuals (n = 12). The expression of SELENOS in omental adipose tissues of T2DM patients was higher than that in non-DM individuals. The Pearson correlation analysis results confirmed that the expression of SELENOS in omental adipose tissues positively correlated with SAA and HOMA-IR [68], suggesting that SELENOS expression in visceral adipose tissues participated in the insulin resistance process in T2DM patients. Previous experimental results in 3T3-L1 adipocytes were mainly obtained in in vitro study conditions in the presence of glucose alone without insulin or in the presence of insulin alone without glucose [14]. These conditions were different from the complicated microenvironment in the body with the co-existence of glucose and insulin, which may be an important reason for the inconsistencies between their study results and results from the studies by Karlsson et al. and our research group. The differences in the comparative results of SELENOS expression in adipose tissues in DM and non-DM populations between the study results by Karlsson et al. and our research group may be due to the differences between subcutaneous adipose tissue and visceral adipose tissue and the compositions of the samples in these two studies. The subjects in the study by Karlsson et al. were all males, while almost 70% of the subjects in our study were females. In addition, the sample sizes in these two studies were 21 and 22 cases (<50 cases); they were both studies with small sample sizes. It is necessary to increase the sample size to investigate the difference of SELENOS expression in adipose tissue between DM and non-DM populations.

#### Skeletal muscle

The contradiction of the association of adipose tissue SELENOS with glucose metabolism in the aforementioned studies was also present in related studies on the association of skeletal muscle SELENOS with glucose metabolism. Walder et al. [14] confirmed that SELENOS expression was inhibited by glucose or insulin in a concentration-dependent manner in in vitro cultured C2C12 muscle cells, while the clinical study of Karlsson et al. [67] showed that the SELENOS mRNA expression in skeletal muscle between T2DM patients and healthy individuals was not different; the differences in the aforementioned in vivo and in vitro microenvironments may be one of the important factors resulting in this inconsistency. In addition, Karlsson et al. [67] confirmed that the SELENOS expression in skeletal muscle positively correlated with the serum SAA level.

SAA can interact with SELENOS, which was confirmed by both a yeast-two hybrid experiment and surface plasmon resonance analysis [14], and the serum SAA level in T2DM patients with short disease courses (≤1 year) was higher than that in a healthy control group [69]. Therefore, these results suggested that SAA may play an important role in the occurrence and development of DM. Subsequently, Marzi et al. [70] performed a 7-year prospective study to show that the high SAA concentration in serum could increase the risk of T2DM by 1.28-fold. Therefore, it can be speculated that SAA may be involved in the interaction of SELENOS expressed in liver, adipose tissue, and skeletal muscle with DM and insulin resistance. In addition, the SELENOS promoter region contained an NF-κB binding site [35]. As a target molecule of NF-κB, SELENOS in liver, adipose tissue, and skeletal muscle may participate in the occurrence and development of DM and insulin resistance through the NF-κB signaling pathway.

#### Pancreas

The progressive apoptosis of pancreatic islet β cells is an important feature of the occurrence and development of DM, and oxidative stress damage induced by high glucose is one of the reasons for pancreatic islet β cell apoptosis. The study of Gao et al. [34] showed that in vitro induction of high SELENOS expression in Min6 pancreatic islet cells could increase the resistance to H_2_O_2_ damage and enhance cell viability, suggesting that SELENOS was a protective factor in pancreatic islet and could protect pancreatic islet β cells from oxidative stress damage. The study by Christensen et al. [18] showed that the selenosulfide bond between ^174^Cys and ^188^Sec in the molecular structure of SELENOS rendered SELENOS with reductase activity; in addition, the study by Liu et al. [51] confirmed that this reductase activity depended on the Trx function, indicating that SELENOS was a Trx-dependent reductase. These results elucidated the possible mechanism underlying the antioxidant protection function of SELENOS on pancreatic islet β cells.

#### Serum

In addition to the localization of SELENOS in the plasma membrane, secreted SELENOS was also detected in the culture medium of HepG2 liver cells and human serum samples. However, secreted SELENOS was not detected in the culture medium of 3T3-L1 adipocytes, L6 skeletal muscle cells, Min6 pancreatic islet β cells, HEK293 human embryonic kidney cells, HUVECs, or human aortic vascular smooth muscle cells (HA/VSMCs), suggesting that serum SELENOS was mainly secreted by hepatocytes [32, 33]. In addition, Gao et al. [32] analyzed the association between secreted SELENOS and glucose metabolism. They detected serum SELENOS in some healthy individuals (27.8 ng/ml), T1DM patients (34.0 ng/ml), and T2DM patients (34.3 ng/ml), with detection rates of 41.7, 23.2 and 27.9%, respectively; however, the mean levels of SELENOS among the three groups were not different. Our study group showed that the serum SELENOS level in T2DM patients was lower than that in healthy individuals. The Spearman correlation analysis showed that the serum SELENOS level negatively correlated with blood glucose, and the detection rate of serum SELENOS in the study population was 100% [33]. The study subjects in the study by Gao et al. [32] were Caucasian whites, and they made the SELENOS primary antibody and the SELENOS standard protein solution used in their serum SELENOS ELISA detection system; in contrast, the study subjects in our study were Asians, and the serum SELENOS detection system used was a commercial ELISA detection kit [33]. The above differences in the races and regions of study subjects and the detection methods were the major causes of the inconsistent results between the studies by Gao et al. [32] and our study group. Therefore, the function and clinical values of serum SELENOS still need to be further confirmed and studied in-depth in prospective randomized controlled trials with larger sample sizes.

**Fig. 2:**
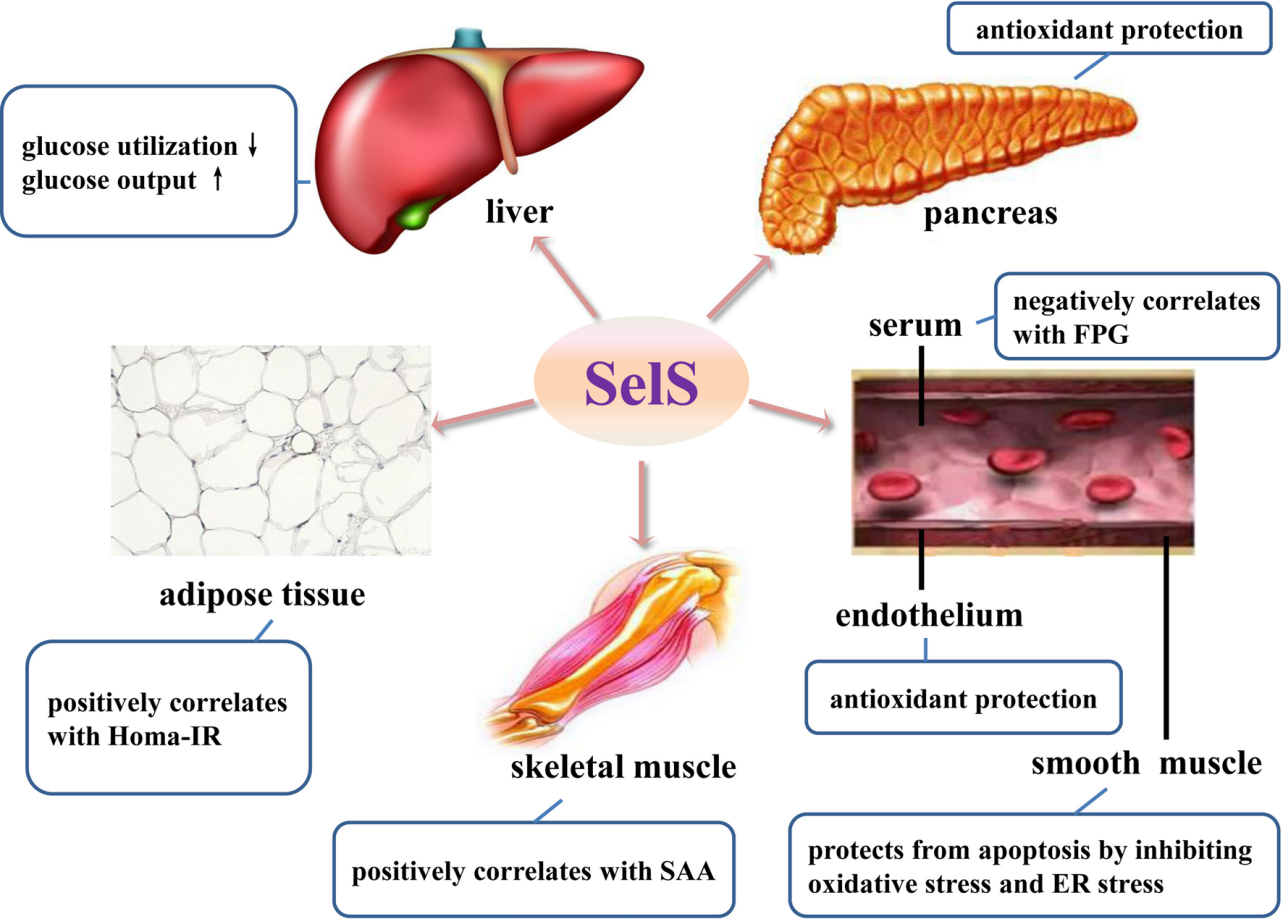
Effects of SELENOS expressed in different tissues and organs on glucose metabolism and macroangiopathy. *HOMA-IR* homeostasis model assessment of insulin resistance, *SAA* serum amyloid A, *ER* endoplasmic reticulum, *FPG* fasting plasma glucose

Therefore, SELENOS in different tissues and organs has different functions. The results are summarized in Fig. 2.

## SELENOS and macrovascular diseases

### Evidence from human genome-wide association studies

Hyrenbach et al. [71] used the Italian and German populations as study subjects and reported that the SELENOS SNP rs28665122 was not associated with ischemic stroke caused by spontaneous cervical artery dissection. In addition, Alanne et al. [72] selected 2222 study subjects from the FINRISK Study in Finland to perform a case-controlled study. They also confirmed that SELENOS polymorphism at that locus was not associated with CVD and ischemic stroke. However, they found that the risk ratio of the development of CVD in females carrying SELENOS SNP rs8025174 was 2.95; the risk ratio of the development of ischemic stroke in the population carrying SNP rs7178239 was 1.75, and the risk ratio in females reached 3.35. Li et al. [73] used the Chinese population as subjects to perform a case-controlled study (ischemic stroke group n = 239; non-ischemic stroke control group n = 240) and showed that SELENOS SNP rs4965814 could increase the risk of ischemic stroke by 1.54-fold; this risk ratio in females reached 2.43. These results were similar to those of Silander et al. [74]. They found that carrying SELENOS SNP rs4965814 increased the risk of ischemic stroke by 2.89-fold in Finnish women; in addition, they also confirmed that carrying SELENOS SNP rs9874 increased the risk of ischemic stroke in Finnish women by 3.32-fold [74]. Furthermore, Cox et al. [75] used European American T2DM patients (n = 1220) as study subjects in the Diabetes Heart Disease Study to analyze the relationship between 10 types of SELENOS gene polymorphism and the risk of atherosclerosis in T2DM patients. The SELENOS SNPs rs28665122, rs4965814, rs28628459, rs7178239, and rs12917258 were associated with subclinical atherosclerosis, and SELENOS SNPs rs4965814, rs28628459, and rs9806366 were associated with clinical atherosclerosis. The above studies suggest that SELENOS gene polymorphism is expected to become one of the indicators for the evaluation of the risk of macroangiopathy in non-DM and T2DM patients and one of the theoretical bases for the adoption of the early enhanced primary prevention measure for macroangiopathy complications in the population carrying relevant SNPs (Table 1).

### Evidence from in vivo and in vitro studies

Vascular endothelial cells play an important role in the maintenance of cardiovascular homeostasis, while endothelial dysfunction is the initiating step in the development of atherosclerosis [76–78]. Our study group induced high/low SELENOS expression in HUVECs in vitro and showed that high SELENOS expression could increase the viability of HUVECs and SOD activity and reduce MDA production stimulated by H_2_O_2_; the changes after inhibition of SELENOS expression had the opposite results [41]. These results indicated that SELENOS was a protective factor in vascular endothelial cells and could increase the resistance of HUVECs to oxidative stress damage.

VSMCs are important components of the tunica media of vascular walls. Their abnormal proliferation is involved in the formation of early atherosclerosis plagues, and the apoptosis of VSMCs in advanced atherosclerosis plaques will induce the reduction of collagen production to cause fibrous cap thinning and reduce plague stability [79], which is a risk factor for cardiovascular events. Recent studies discovered that inhibition of SELENOS expression in VSMCs could aggravate cell damage caused by H_2_O_2_ or TM and increase VSMC apoptosis. Therefore, it was speculated that SELENOS could increase the resistance of VSMCs to oxidative stress and ER stress [42].

Our study group further showed that high SELENOS expression could inhibit the increase of caveolin-1 (Cav-1) induced by H_2_O_2_. When SELENOS expression was inhibited, Cav-1 and protein kinase Cα (PKCα) expression levels increased [41], suggesting that the protection of HUVECs from oxidative stress damage by SELENOS was associated with the regulation of Cav-1 and PKCα expression. Ye et al. [42] confirmed that inhibition of SELENOS expression in VSMCs further increased the phosphorylation levels of MAPK and JNK stimulated by H_2_O_2_, indicating that SELENOS increased the resistance of VSMCs to oxidative stress damage through the MAPK/JNK pathway. In addition, the antioxidant function of SELENOS in blood vessels may also be associated with the aforementioned ^188^Sec in its molecular structure. Furthermore, the study of Liu et al. [51] also showed that SELENOS had peroxidase activity and could break down H_2_O_2_ into H_2_O, which could partially explain the study results of our group [41] and those of Ye et al. [42]. These results elucidated the possible mechanism underlying the macrovascular protective function of SELENOS (Fig. 2).

## SELENOS and other diseases

### SELENOS and cancer

Shibata et al. [80] confirmed that carrying SELENOS SNP rs28665122 was associated with a 1.99-fold increased risk of intestinal type gastric cancer in Japanese individuals. Mao et al. [81] also showed that the risk ratio of gastric cancer in the population carrying SELENOS SNP rs34713741 was 1.62. The studies of Méplan et al. [82] and Sutherland et al. [83] confirmed that this variation could increase the risk of colorectal cancer by 1.68- and 1.57-fold, respectively, and the risk ratios in women increased to 1.83 and 2.25, respectively. In addition, Hart et al. [84] showed that a combination of gene variations in SELENOS SNP rs28665122, caspase 8, matrix metalloproteinase 1 (MMP 1), and IL-10 increased the risk of the development of non-small cell lung cancer in the Norwegian population by 4.62-fold. The above studies suggest that the SELENOS gene polymorphism is expected to become one of the indicators for the risk of cancer. The association between SELENOS gene variation and the risk of cancer and the associations between SELENOS gene variation combined with other gene variations and the risk of cancer still require more studies for investigation and validation. In addition, there is currently no in vivo and in vitro functional study of SELENOS in the occurrence and development of cancer (Table 1).

### SELENOS and autoimmune inflammatory diseases

It has been confirmed that various SELENOS SNPs are associated with the risk of the development of autoimmune inflammatory diseases. Marinou et al. [85] showed that the co-presence of SELENOS SNP rs28665122 and IL-1β SNP rs16944 increased the risk of development of rheumatoid arthritis in Caucasians by 2.3-fold. Santos et al. [86] showed that carrying SELENOS SNP rs28665122 could increase the risk of Hashimoto’s thyroiditis in Portuguese by 2.22-fold. This conclusion was subsequently confirmed by Li et al. [87] in a case-controlled study using the Chinese population as their study subjects. They discovered that the risk ratio of the development of Hashimoto’s thyroiditis in the population carrying SELENOS SNP rs28665122 was 1.28. In addition, SELENOS SNP rs28665122 has also been confirmed to be associated with preeclampsia, spontaneous preterm birth, and Kashin–Beck disease (KBD) [88–90]. The study by Du et al. [90] showed that the expression of PI3K and Akt in the whole blood of carriers with that variation was higher than that of non-carriers and that the expression levels of PI3K and Akt were higher in the whole blood of KBD patients compared with the control group. Therefore, it was speculated that SELENOS SNP rs28665122 participated in the development of KBD through the regulation of PI3K/Akt pathway (Table 1).

## Conclusions and prospects

SELENOS is closely associated with inflammation, oxidative stress, and ER stress. In-depth and comprehensive understanding of its association with the above reactions may not only elucidate or supplement pathogenic mechanisms of relevant diseases but also provide new evidence for clinical physicians to develop new strategies for disease prevention and treatment. However, the current understanding of this aspect is limited; there are still some scientific questions that need to be further confirmed or studied in-depth, such as whether SELENOS can inhibit the expression of other inflammatory factors and whether the regulation of ER stress by SELENOS is different in different cell types. Furthermore, whether the human SELENOS gene promoter region has negative regulatory elements that can regulate SELENOS gene expression and whether there are transcription factors that can interact with these elements should also be further explored. These studies will help to provide new targets for drug intervention and to discover more effective disease treatment methods.

The functions of SELENOS in inflammatory reactions, oxidative stress, and ER stress point to its great potential in DM and macroangiopathy. It has been shown that SELENOS expressed in different tissues and organs has different effects on the occurrence and development of DM and its macroangiopathy. The high expression of SELENOS in pancreatic islets and blood vessels can exert an antioxidant protection function and can increase the defense capacity of VSMCs to ER stress, while SELENOS expressed in liver, adipose tissue, and skeletal muscle can promote the occurrence and development of DM and insulin resistance. Based on the above functional characteristics of SELENOS, the method by which tissues and organs specifically regulate its expression to fully utilize the advantages of SELENOS and weaken its adverse effects still require further in-depth studies. SELENOS gene polymorphism is associated with DM, macroangiopathy, tumors, and autoimmune inflammatory diseases; therefore, it is expected to become one of the indicators for the prediction of the risk of the above diseases and one of the theoretical bases for the adoption of primary preventive measures for the population carrying relevant SNPs. However, SELENOS SNPs that have been discovered to be associated with disease development must still be confirmed in different populations using larger sample sizes.

## Abbreviations

ER: endoplasmic reticulum
DM: diabetes mellitus
SELENOS: selenoprotein S
WHO: World Health Organization
CVD: cardiovascular disease
Sec: selenocysteine
GPxs: glutathione peroxidases
DIs: iodothyronine deodinases
TRs: thioredoxin reductases
SECIS: selenocysteine insertion sequence
3′UTR: 3′untranslated region
CDS: coding domain sequence
SAA: serum amyloid A
LPS: lipopolysaccharide
IL-1β: interleukin-1β
IL-6: interleukin-6
TNFα: tumor necrosis factor α
MCP 1: monocyte chemoattractant protein 1
IL-2: interleukin-2
ERSE: ER stress-response element
SNP: single nucleotide polymorphism
IL-10: interleukin 10
G-CSF: granulocyte colony-stimulating factor
Trx: thioredoxin
H_2_O_2_: hydrogen peroxide
HUVECs: human umbilical vein endothelial cells
SOD: superoxide dismutase
MDA: methane dicarboxylic aldehyde
GSH: glutathione
ROS: reactive oxygen species
VSMCs: vascular smooth muscle cells
MAPK: mitogen-activated protein kinase
JNK: c-JUN N-terminal kinase
Derlin1: degradation in endoplasmic reticulum protein 1
ERAD: ER-associated protein degradation
TM: tunicamycin
TG: thapsigargin
GRP78: glucose-regulated protein 78
XBP-1: X-box binding protein1
CHOP: CCAAT/enhancer-binding protein homologous protein
HOMA-IR: homeostasis model assessment of insulin resistance
PEPCK: phosphoenolpyruvate carboxykinase
Cav-1: caveolin-1
PKCα: protein kinase Cα
MMP 1: matrix metalloproteinase 1
KBD: Kashin–Beck disease
